# Estimated Prevalence of Metastatic Breast Cancer in England, 2016-2021

**DOI:** 10.1001/jamanetworkopen.2022.48069

**Published:** 2022-12-22

**Authors:** Carlo Palmieri, Joshua Owide, Kirsty Fryer

**Affiliations:** 1 Department of Molecular and Clinical Cancer Medicine, Institute of Systems, Molecular and Integrative Biology, University of Liverpool, Liverpool, United Kingdom; 2 The Clatterbridge Cancer Centre NHS Foundation Trust, Liverpool, United Kingdom; 3 Wilmington Healthcare Ltd, London, United Kingdom

## Abstract

This case series study provides an estimate of total cases of metastatic breast cancer in England based on National Health Service data.

## Introduction

In England, there were 48 387 new cases of breast cancer with 9525 breast cancer recorded deaths in 2019.^1^ The vast majority of these breast cancer deaths were due to metastatic breast cancer (MBC). However, data on the prevalence of MBC within England is not collected or reported, contributing to a number of important data gaps. An Australian study estimated that there are 3 to 4 prevalent cases of metastatic breast cancer per breast cancer death.^2^ Meanwhile, data from the Surveillance, Epidemiology and End Results (SEER) registries estimated 154 794 women would be living with MBC in the US by January 2017.^3^ Both studies highlighted the increasing number of prevalent cases of MBC over time,^2,3^ with a further study estimating that by 2030 there would be 246 194 prevalent cases in the US.^4^ Given the lack of published English data, we sought to identify the prevalent MBC population in England, how it had changed over the preceding 5 years, and the clinical activity generated by this population within the National Health Service (NHS) in England.

## Methods

This population-based case series utilized secondary care records extracted from the English Hospital Episode Statistics (HES) database held by NHS England; data were produced by NHS Digital, the new trading name for the Health and Social Care Information Centre (HSCIC), and reused with the permission of NHS Digital. Access to licensed HES data was provided through Wilmington Healthcare; the study was exempted from ethics approval after review for data access from the Independent Group Advising on the Release of Data for the NHS digital board (eMethods in the Supplement). This study followed the reporting guideline for case series.

Data were extracted for the 5 financial years they were accessible and licensed for use by NHS Digital, April 1, 2016, to March 31, 2021. Cases of MBC were identified based on a primary diagnosis of breast cancer and any combination of a metastatic cancer diagnoses identified utilizing codes from the *International Statistical Classification of Diseases and Related Health Problems, Tenth Revision* (*ICD-10*) (eMethods in the Supplement).

## Results

**Figure 1.**
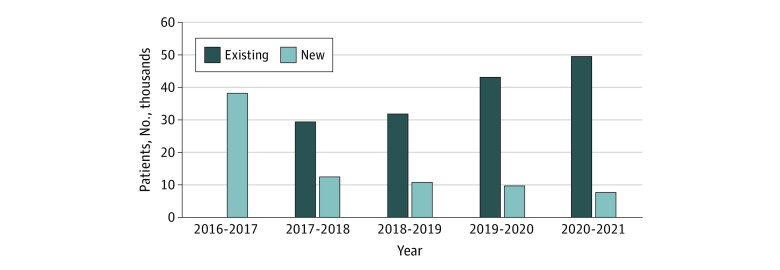
Prevalent Population With Metastatic Breast Cancer in England Identified From Hospital Episode Statistics by Financial Year

**Figure 2.**
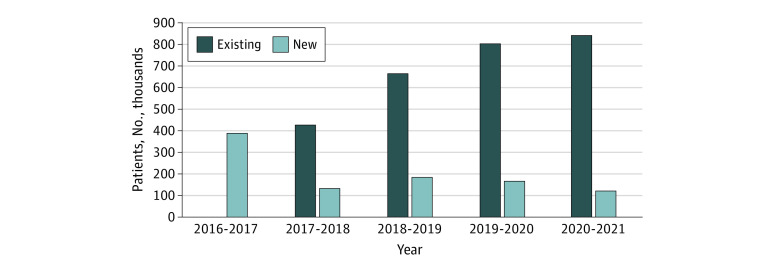
Total Hospital Spells Recorded for the Prevalent Population With Metastatic Breast Cancer in England by Financial Year

We identified a prevalent population of 57 215 patients with MBC in England for the financial year running from April 1, 2020, to March 31, 2021 (Figure 1). In the preceding 4 financial years (2016-2017, 2017-2018, 2018-2019, and 2019-2020) the identified prevalent MBC population was 38 350, 41 985, 43 015, and 52 755, respectively. For the four 4 years (2017-2018 to 2020-2021) when the number of new patients with metastatic breast cancer could be identified, this varied between 7580 (2020-2021) to 12 540 (2017-2018) new patients per financial year (Figure 1). In terms of hospital spells generated by the prevalent metastatic population for 2020-2021, a total of 974 320 spells of activity were recorded. Overall, the number of hospital spells increased over the 5 years that data was available from 393 180 (2016-2017) to 974 320 (2020-2021) (Figure 2).

## Conclusions

We used HES data, which cover patients treated by the NHS in England, to identify patients with MBC treated between April 2016 and March 2021. HES data were also used to identify the number of hospital spells for this prevalent population. To our knowledge, this is the first time that the number of people living with MBC in England has been estimated. The number of new cases per year and the clinical activity generated is also reported for the first time. Estimates have suggested that 35 000 individuals are living with MBC in the UK.^5^ However, the current estimate of over 57 000 in England alone indicates that this is an underestimation. The data demonstrates, in keeping with Australia and US data, that the prevalent population with MBC is increasing over time. The 974 320 hospital episodes for the financial year 2020-2021 provides an indication of the level of clinical activity and work generated by patients with MBC, and again with an upward trend over time.

Study limitations include that, because HES data does not contain pathology or treatment information, we were unable to report the nature of the hospital episode or the number of deaths. Given *ICD-10* codes for metastatic cancer were used, there is a possibility some patients with a relapsed second primary may have been captured. Our findings are important as they provide data on the prevalence of MBC using NHS data. Such data are key as they provide MBC patients with visibility, as well as being vital for ensuring the design, commissioning and delivery of appropriate services with adequate capacity.

## Notes

Supplement.
eMethods.
