# Supplementary material for: Estimated Prevalence of Metastatic Breast Cancer in England, 2016-2021
Source: JAMA Netw Open. 2022 Dec 22;5(12):e2248069. doi: 10.1001/jamanetworkopen.2022.48069 (PMC9857429; doi:10.1001/jamanetworkopen.2022.48069)
Supplement: Supplement. — eMethods. [file jamanetwopen-e2248069-s001.pdf]

Supplemental Online Content

Palmieri C, Owide J, Fryer K. Estimated prevalence of metastatic breast cancer in England, 2016-2021. *JAMA Netw Open.* 2022;5(12):e2248069.
doi:10.1001/jamanetworkopen.2022.48069

eMethods.

This supplemental material has been provided by the authors to give readers additional information about their work.

© 2022 Palmieri C et al. *JAMA Network Open.*

eMethods. Process for data Access

Wilmington Healthcare were granted access to English Hospital Episode Statistics (HES) data following an application to the Data Access Request Service (DARS). This request was reviewed and approved by the Independent group advising on the release of data (IGARD), an advisory body to the NHS digital board. No ethics approval was therefore required.**Identification of prevalent population**

The following codes were used for primary breast cancer:

C500 - Malignant neoplasm: Nipple and areola

C501 - Malignant neoplasm: Central portion of breast

C502 - Malignant neoplasm: Upper-inner quadrant of breast

C503 - Malignant neoplasm: Lower-inner quadrant of breast

C504 - Malignant neoplasm: Upper-outer quadrant of breast

C505 - Malignant neoplasm: Lower-outer quadrant of breast

C506 - Malignant neoplasm: Axillary tail of breast

C508 - Malignant neoplasm: Overlapping lesion of breast

C509 - Malignant neoplasm: Breast, unspecified

These were combined with the following metastatic cancer codes:

C770 - Secondary and unspecified malignant neoplasm: Lymph nodes of head, face and neck

C771 - Secondary and unspecified malignant neoplasm: Intrathoracic lymph nodes

C772 - Secondary and unspecified malignant neoplasm: Intra-abdominal lymph nodes

C773 - Secondary and unspecified malignant neoplasm: Axillary and upper limb lymph nodes

C774 - Secondary and unspecified malignant neoplasm: Inguinal and lower limb lymph nodes

C775 - Secondary and unspecified malignant neoplasm: Intrapelvic lymph nodes

C778 - Secondary and unspecified malignant neoplasm: Lymph nodes of multiple regions

C779 - Secondary and unspecified malignant neoplasm: Lymph node, unspecified

C780 - Secondary malignant neoplasm of lung

C781 - Secondary malignant neoplasm of mediastinum

© 2022 Palmieri C et al. *JAMA Network Open.*

C782 - Secondary malignant neoplasm of pleura

C783 - Secondary malignant neoplasm of other and unspecified respiratory organs

C784 - Secondary malignant neoplasm of small intestine

C785 - Secondary malignant neoplasm of large intestine and rectum

C786 - Secondary malignant neoplasm of retroperitoneum and peritoneum

C787 - Secondary malignant neoplasm of liver and intrahepatic bile duct

C788 - Secondary malignant neoplasm of other and unspecified digestive organs

C790 - Secondary malignant neoplasm of kidney and renal pelvis

C791 - Secondary malignant neoplasm of bladder and other and unspecified urinary organs

C792 - Secondary malignant neoplasm of skin

C793 - Secondary malignant neoplasm of brain and cerebral meninges

C794 - Secondary malignant neoplasm of other and unspecified parts of nervous system

C795 - Secondary malignant neoplasm of bone and bone marrow

C796 - Secondary malignant neoplasm of ovary

C797 - Secondary malignant neoplasm of adrenal gland

C798 - Secondary malignant neoplasm of other specified sites

C799 - Secondary malignant neoplasm, unspecified site

Individual patients with a relevant metastatic breast cancer diagnosis were classified as either new or existing in each financial year based on when they first appeared in the HES dataset with a relevant metastatic breast cancer diagnosis. A distinct patient count was subsequently generated to establish the overall 12 month prevalence of metastatic breast cancer by financial year. Given data was not available for the financial year which ran from the 1st April 2015-to 31st March 2016 it was not possible to differentiate new versus existing for the subsequent financial year,the 1st April 2016 to 31st March 2017. Data relates to admissions for inpatient care,andappointments for outpatient care within English healthcare facilities, these were combined to generate an overall activity metric for the cohort

© 2022 Palmieri C et al. *JAMA Network Open.*

of metastatic breast cancer patients by financial year. The STROBE reporting guideline for observational studies were followed.

© 2022 Palmieri C et al. *JAMA Network Open.*

1. To document the prevalence and incidence of metastatic breast cancer.

2. To measure outcomes both overall and by key breast cancer sub-types.

3. To look at disparities in diagnosis, care and treatments eg access to specialist nurses, discussion at MDTs and treatments

4. To design and configuration of modern services based on the population and their needs.

5. To know what our workforce should look like eg number of specialist secondary breast cancer nurses required.

6. To identify important clinical questions that need addressing

7. To aid design and delivery of clinical trials.

8. To encourage engagement with life sciences industry and pharma and so attracting investment for clinical and translational studies.

9. Visibility for patients and enabling advocacy

© 2022 Palmieri C et al. *JAMA Network Open.*
